# Association between Vitamin D Insufficiency and Elevated Serum Uric Acid among Middle-Aged and Elderly Chinese Han Women

**DOI:** 10.1371/journal.pone.0061159

**Published:** 2013-04-09

**Authors:** Hao Peng, Hongmei Li, Chao Li, Xiangqin Chao, Qiu Zhang, Yonghong Zhang

**Affiliations:** 1 Department of Epidemiology, School of Public Health, Medical College of Soochow University, Suzhou, China; 2 Department of Medicine, University of Hong Kong, Queen Mary Hospital, Hong Kong, China; 3 Center for Disease Prevention and Control of Jinchang District, Suzhou, China; Baylor College of Medicine, United States of America

## Abstract

**Background:**

Association between vitamin D insufficiency and hyperuricemia has not been reported so far. We aimed to study the association of vitamin D insufficiency with elevated serum uric acid among middle-aged and elderly Chinese Han women.

**Methods:**

We collected data from participants residing in Jinchang district of Suzhou from January to May, 2010. Serum uric acid, 25-hydroxy vitamin D and other traditional biomarkers including fasting plasma glucose and blood lipids were determined in 1726 women aged above 30 years. Association between vitamin D insufficiency and elevated uric acid was analyzed in premenopausal and postmenopausal women, respectively.

**Results:**

Among postmenopausal women, 25-hydroxy vitamin D level of participants with elevated uric acid was lower than that of those with normal uric acid (median [interquartile range]: 35[28–57] *vs* 40[32–58], µg/L; *P* = 0.006). Elevated uric acid was more prevalent in participants with vitamin D insufficiency compared to those without vitamin D insufficiency (16.50% *vs* 8.08%; *P*<0.001). Association between vitamin D insufficiency and elevated uric acid was not significant among premenopausal women. However, participants with vitamin D insufficiency were more likely to have elevated uric acid compared with those without vitamin D insufficiency among postmenopausal women (OR, 95% CI: 2.38, 1.47–3.87). Moreover, after excluding individuals with diabetes and/or hypertension, the association of vitamin D insufficiency with elevated uric acid was still significant (OR, 95% CI: 2.48, 1.17–5.44).

**Conclusions:**

Vitamin D insufficiency was significantly associated with elevated uric acid among postmenopausal Chinese Han women. This study suggested that a clinical trial should be conducted to confirm the association of vitamin D insufficiency with hyperuricemia.

## Introduction

Cardiovascular disease (CVD), a global public health burden, is increasingly prevalent [1]. As a predictor of CVD, hyperuricemia has been long observed to occur with an increased frequency among population with high risks for CVD [2], [3]. Moreover, some previous studies found that vitamin D deficiency was an independent risk factor for CVD [4] and a predictor of chronic kidney diseases [5]. Impaired renal function was suggested to raise serum uric acid (SUA) concentration by decreasing renal excretion [6]. Recently, an increasing body of literature has suggested that a significant biologic influence of parathyroid hormone on SUA exists [7], [8]. Meanwhile, vitamin D deficiency or insufficiency can activate parathyroid to induce the release of parathyroid hormone [9]. A previous clinical trial in postmenopausal women indicated that parathyroid hormone increased the incidence of hyperuricemia [10]. All these evidences suggested an inverse association between circulating 25-hydroxy vitamin D (25(OH)D) and elevated SUA. To our knowledge, there were no previous studies reporting the relationship between vitamin D insufficiency and hyperuricemia in Asian population. In addition, there was a gender and age-related discrepancy in vitamin D status [11] and prevalence of hyperuricemia [12]. Therefore, we examined serum 25(OH)D and SUA for 1726 Chinese Han women aged above 30 years in Suzhou and studied the association between vitamin D insufficiency and elevated SUA in premenopausal and postmenopausal women, respectively.

## Materials and Methods

### Study participants

We conducted a cross-sectional study in a traditional but economically developed district of Suzhou from January to May, 2010. The subjects were selected via multiphase cluster random sampling. From the total 20 urban communities and 19 rural villages in the district, 4 urban communities and 4 rural villages were randomly selected as the research fields. The subjects were selected from the 8 selected study fields. The selection criteria were to meet all of the followings: (1) age: ≥30 years, (2) ethnicity: Han, (3) gender: female, (4) no clinical evidence of end-organ damage (no clinical records on angina pectoris, myocardial infarction, heart failure, stroke, hypertensive encephalopathy, retinal hemorrhage, and renal failure) [13] at the screening visit, (5) no chronic renal diseases or tumors. The exclusion criteria were to meet one of the followings: (1) having clinical suspicion of renal calculi, pyelonephritis or urinary stones, (2) having used SUA lowering medication within the last two weeks, (3) self-reported thyroid or parathyroid diseases, (4) being pregnant, (5) self-reported gout or arthritis. There were a total of 2338 eligible women in the study fields, but only 1896 women participated in the study. After exclusion of 170 individuals for lacking urine or blood samples, 1726 subjects were finally eligible for this study. This study was approved by the Soochow University Ethics Committee. Written informed consent was obtained from all study participants.

### Data collection

Data on demographic information, lifestyle risk factors, and personal medical history were gathered from standard questionnaires in Chinese language administered by trained staff. Cigarette smoking was defined as having smoked at least 1 cigarette per day for 1 year or more and reported current smoking. Alcohol consumption was defined as consuming any type of alcohol beverage at least once per week during the last three years. Body weight and height were measured using a regularly calibrated stadiometer and balance-beam scale with participants wearing light clothing and no shoes. Body mass index (BMI) was calculated as weight in kilograms divided by height in meters squared. Waist circumference (WC) was measured at the level of 1 cm above the umbilicus. Three consecutive sitting blood pressure measurements (3 minutes between each) were taken by trained staff using a standard mercury sphygmomanometer according to a standard protocol, after the subjects had been resting for 30 minutes [13]. The first and fifth Korotkoff sounds were recorded as systolic blood pressure (SBP) and diastolic blood pressure (DBP), respectively. The mean of the three records was used in analysis. Hypertension was defined as SBP ≥140 mmHg and/or DBP ≥90 mmHg and/or use of antihypertensive medication in the last two weeks [13]. Diabetes was defined as fasting plasma glucose (FPG) ≥7.0 mmol/L and/or use of hypoglycemic medication in the last two weeks [14].

Blood samples were obtained by venipuncture in the morning after a requested overnight fast (at least 8 hours). All plasma and serum samples were frozen at −80°C until laboratory testing. Total cholesterol (TC), triglycerides (TG), FPG and SUA were measured for all subjects. All the biochemical indexes were analyzed enzymatically on Hitachi 7020 automatic biochemical analyzer using commercial reagents (KANGXIANG MEDICAL APPliANCE, Shanghai, P.R China). Intra- and inter-assay coefficients of variation were less than 2% and 4%, respectively.

Serum 25(OH)D level is a reliable indicator to assess vitamin D insufficiency, adequacy, or toxicity. Serum 25(OH)D measurements were performed in Thermo Scientific Multiskan Spectrum microplate reader (Thermo Fisher Scientific Inc., Waltham, Massachusetts, USA) by ELISA method applying IBL antibodies (Immuno-Biological Laboratories Co., LTD. Gunma, JAPAN). All of the samples were processed in a duplicate assay. A standard curve was constructed and from which the 25(OH)D concentrations of unknown samples were determined. Intra- and inter-assay coefficients of variation were less than 9% and 11%, respectively. As for vitamin D status, insufficiency was defined as a serum 25(OH)D <30 ug/L and sufficiency was defined as a serum 25(OH)D ≥30 ug/L [11].

### Statistical analysis

Statistical analysis was conducted using SAS statistical software (version 9.1, Cary, North Carolina, USA). Period from 45 to 55 years is the most common menopausal transition in Chinese Han women [15]. Therefore, we studied individuals younger and older than 55 years, respectively, due to the metabolic influence of estrogen [11], [12]. In current analysis SUA above the 90th percentile was considered to be elevated in each individual age-group. Baseline characteristics of participants with and without elevated SUA in each individual age-group were compared. Comparisons in means of continuous variables with a normal distribution were performed by using Student's t-test. Comparisons in medians of continuous variables with a skewed distribution were performed by using a Wilcoxon rank-sum test. Comparisons in rates for categorical variables were performed by using the Chi-square test. The prevalence of elevated SUA in participants with and without vitamin D insufficiency was computed and compared using the Chi-square test. For skewed distribution, medians of 25(OH)D in the two groups were compared using a Wilcoxon rank-sum test. A Pearson correlation analysis was used to estimate correlation between log-transformed 25(OH)D and SUA values in premenopausal and postmenopausal women, respectively. Univariate and multivariate non-conditional logistic regression models were used to assess association between 25(OH)D levels and risk of elevated SUA. In current analysis, odds ratio (OR) and 95% confidence interval (CI) of elevated SUA was estimated for participants with vitamin D insufficiency compared to those with vitamin D sufficiency. Furthermore, participants were categorized into quartiles of 25(OH)D values in premenopausal and postmenopausal women, respectively. Elevated SUA was the dependent variable while 25(OH)D quartiles and other covariates were independent variables in the regression models. ORs and 95% CI of elevated SUA were calculated for lower quartiles of 25(OH)D with the highest quartile as a reference. Trends in the ORs of elevated SUA across decreasing 25(OH)D categories were determined, modeling 25(OH)D category as an ordinal variable. Variables that significantly differed between participants with and without vitamin D insufficiency were used as covariates in the multivariate models. Increasing findings from meta-analysis suggested that both hypertension and diabetes were influenced by vitamin D statuses [16], [17] and SUA levels [18], [19]. Therefore, in order to minimize or even eliminate the impact of hypertension and diabetes on the association between vitamin D and SUA, participants were further categorized into two sub-groups in each age-group: individuals with either, both or none of hypertension and diabetes. The multivariate logistic regression models were repeated in each sub-group. A two-tailed *P* value <0.05 was considered statistically significant.

## Results

### Baseline characteristics of premenopausal and postmenopausal women

Among 1726 women (54.00±10.39 years) included in current analysis, 858 (49.71%) participants aged below 55 years were considered to be premenopausal and 868 (50.29%) participants aged over 55 years were considered to be postmenopausal. 86 premenopausal women were considered to have elevated SUA (greater or equal to 314 umol/L, the 90th percentile of SUA in the premenopausal women) and 87 postmenopausal women were considered to have elevated SUA (greater or equal to 357 umol/L, the 90th percentile of SUA in the postmenopausal women). As shown in Table 1, women with elevated SUA had higher levels of FPG, TG, BMI, SBP and DBP and larger WC than those with normal SUA level among both premenopausal and postmenopausal women (all *P*<0.05). TC level in individuals with elevated SUA was higher than that in participants with normal SUA among premenopausal women, but there was no significant difference in TC level between the two groups among postmenopausal women than in those with normal SUA. Moreover, both hypertension and diabetes were more common among premenopausal women with elevated SUA compared with those with normal SUA level (all *P*<0.05). And only hypertension was more common in postmenopausal women with elevated SUA (*P*<0.001). In postmenopausal women, participants with elevated SUA were more likely to have vitamin D insufficiency (*P* = 0.001). However, there was no significant difference in serum 25(OH)D median level or vitamin D insufficiency prevalence between premenopausal women with and without elevated SUA.

**Table 1:** Baseline characteristics of participants with and without elevated uric acid by different age-groups.

Characteristics	Age <55 years		*P* value	Age ≥55 years		*P* value
	Normal SUA	Elevated SUA		Normal SUA	Elevated SUA	
NO. of participants	772	86	-	781	87	-
Suburb residents, n (%)	220(28.50)	17 (17)	0.086	360(46.09)	41(47.13)	0.855
Smoker, n (%)	6(0.78)	0(0.00)	1.000	3(0.38)	1(1.15)	0.345
Drinker, n (%)	23(2.98)	3 (3.49)	0.739	21(2.69)	2(2.30)	1.000
Education (years)						
0–6, n (%)	216 (27.98)	31 (36.05)	0.080	523 (66.97)	58(66.67)	0.907
7–9, n (%)	361 (46.76)	39 (45.35)		150(19.21)	19(21.84)	
≥10, n (%)	195 (25.26)	16 (18.60)		108(13.83)	10(11.49)	
FPG, mmol/L	5.0(4.6–5.5)	5.2(4.8–5.7)	0.004	5.3(4.9–5.9)	5.6(5.0–6.2)	0.024
TG, mmol/L	0.90(0.67–1.28)	1.34(1.06–2.21)	<0.001	1.20(0.88–1.70)	1.60(1.17–2.29)	<0.001
TC, mmol/L	4.82(4.33–5.40)	5.31(4.62–5.85)	0.0002	5.46(4.87–6.09)	5.59(5.0–6.49)	0.071
BMI, kg/m2	23.6(21.8–25.7)	25.5(23.5–27.1)	<0.001	24.6(22.7–27.0)	25.9(23.7–28.4)	0.001
WC, cm	77.5(72.0–82.8)	81.5(75.5–86.0)	0.0002	82(76.0–88.3)	86.7(80.1–93.0)	<0.001
SBP, mmHg	122(114–133)	128(118–136)	0.013	134(124–144)	143(133–155)	<0.001
DBP, mmHg	82(76–86)	84(80–88)	0.005	84(80–89)	85(81–93)	0.036
Hypertension, n (%)	190(24.61)	32(37.21)	0.011	413(52.88)	70(80.46)	<0.001
Diabetes, n (%)	31(4.02)	8(9.30)	0.026	75(9.60)	10(11.49)	0.574
25(OH)D, µg/L	40(33–51)	41(36–54)	0.364	40(32–58)	35(28–57)	0.006
25(OH)D <30 µg/L, n (%)	133(17.23)	14(16.28)	0.825	167(21.38)	33(37.93)	0.001

All values are expressed with median (inter-quartile range) unless otherwise noted. SUA, serum uric acid; FPG, fasting plasma glucose; TG, triglycerides; TC, total cholesterol; BMI, body mass index; WC, waist circumference; SBP, systolic blood pressure; DBP, diastolic blood pressure; 25(OH)D, 25- hydroxy vitamin D.

### Prevalence of elevated SUA in participants with and without vitamin D insufficiency

**Figure 1:**
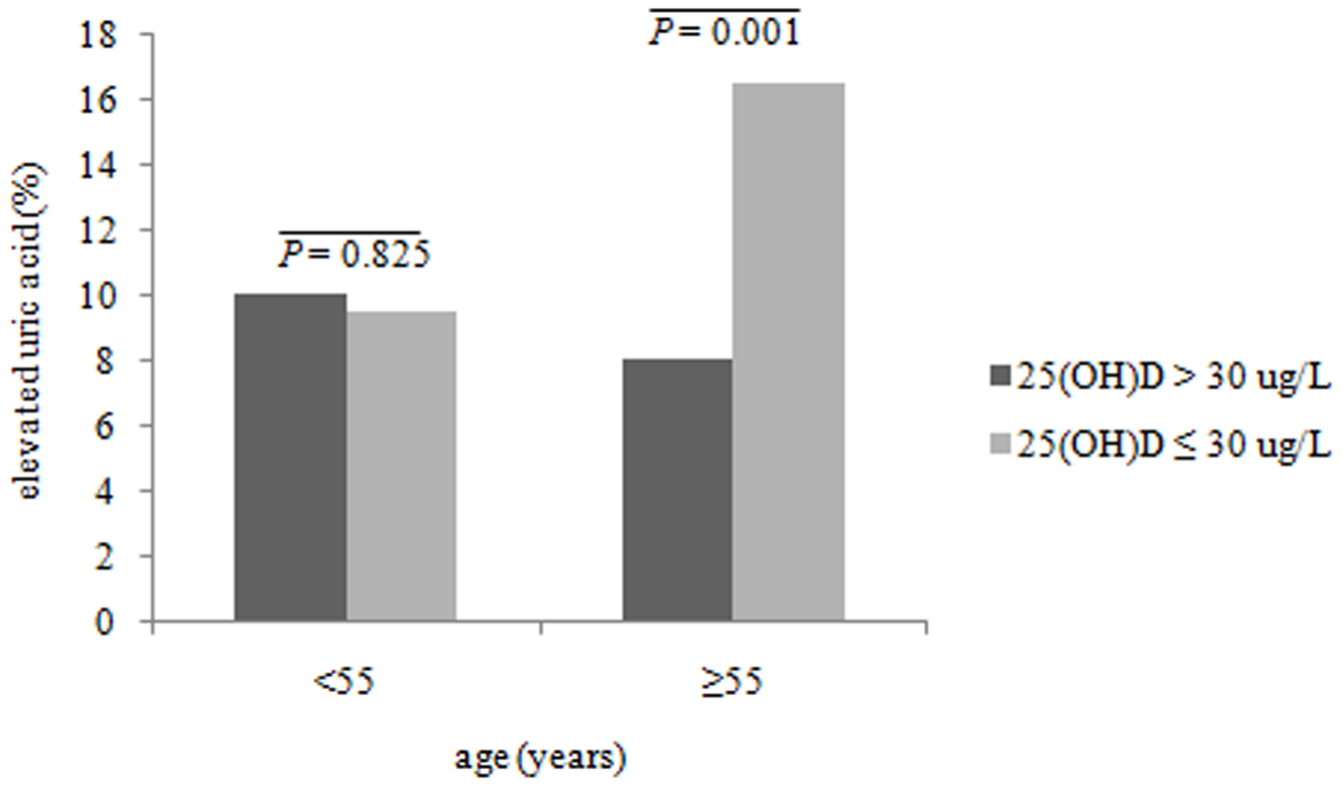
Prevalence of elevated uric acid in individuals with and without vitamin D insufficiency by different ages.

We further compared the prevalence of elevated SUA between participants with and without vitamin D insufficiency. As shown in Figure 1, we did not find a significant difference in elevated SUA prevalence between participants with and without vitamin D insufficiency among premenopausal women, with a prevalence of 10.13% and 9.52%, respectively. Among postmenopausal women, elevated SUA was more common in women with vitamin D insufficiency than those with vitamin D sufficiency (*P* = 0.001), with a prevalence of 16.50% in participants with vitamin D insufficiency and 8.08% in those with vitamin D sufficiency.

### Correlation between serum 25(OH)D and SUA

**Figure 2:**
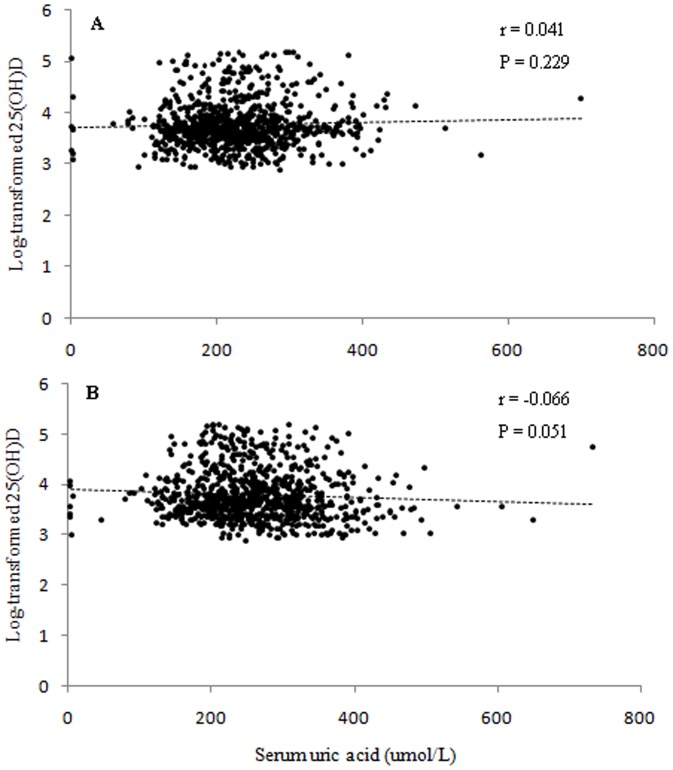
Correlation between log-transformed serum 25-hydroxy vitamin D concentration and uric acid. The correlation between 25-hydroxy vitamin D and uric acid was evaluated among premenopausal (A) and postmenopausal women (B), respectively.

As shown in Figure 2, correlation between 25(OH)D and SUA was evaluated in premenopausal and postmenopausal women after log-transformation of 25(OH)D. No significant correlation between 25(OH)D and SUA was found among premenopausal women. The correlation was in the expected direction (r = −0.066) among postmenopausal women although the *P* value of Pearson correlation analysis was not statistically significant as the *P* value was 0.051.

### Association between vitamin D insufficiency and elevated SUA

**Figure 3:**
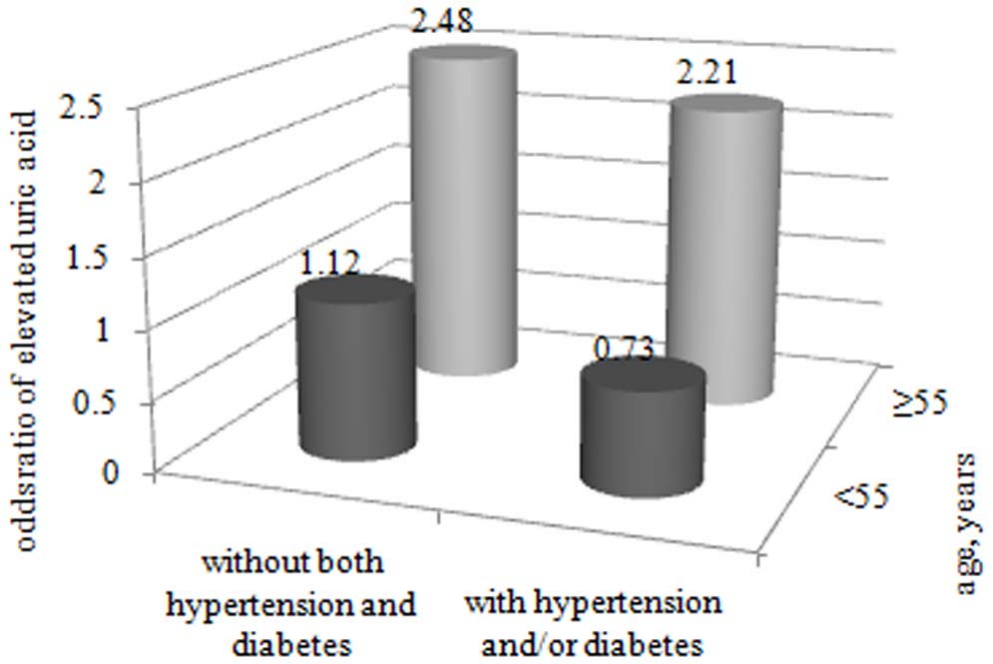
Odds ratio and 95% confidence interval of elevated uric acid for women with vitamin D insufficiency by different ages and disease status. Participants were further categorized into two sub-groups in each age-group: individuals with either, both or none of hypertension and diabetes. The multivariate logistic regression analysis was performed in all sub-groups and odds ratios and 95% confidence interval were computed. Covariates like residential site, education status, cigarette smoking, alcohol drinking, triglycerides and body mass index were adjusted for in the multivariate models.

As shown in Table 2, among premenopausal women, OR of elevated SUA for individuals with vitamin D insufficiency was not significant compared to those with vitamin D sufficiency in either univariate or multivariate models. Whereas, a marked association between vitamin D insufficiency and risk of elevated SUA was observed among postmenopausal women. In univariate analysis, participants with vitamin D insufficiency were more likely to have elevated SUA than those with vitamin D sufficiency (*P* = 0.001). After adjustment for residential site, education status, cigarette smoking, alcohol consumption, TG, FPG, BMI, SBP and DBP, OR of elevated SUA for women with vitamin D insufficiency increased by 1.38-fold than those with vitamin D sufficiency (*P* = 0.001). Although FPG and blood pressure were adjusted for in the multivariate models, sub-group analyses were still essentially needed to eliminate the potential influence of diabetes and hypertension, because the two diseases were found to be closely associated with vitamin D and SUA [16]–[19]. Hence, besides the age-groups, participants were further categorized as women with either, both or none of hypertension and diabetes. The results of the multivariate analysis were shown in Figure 3. After adjusting for residential site, education status, cigarette smoking, alcohol consumption, TG, and BMI, a significant association between vitamin D insufficiency and elevated SUA persisted in both sub-groups among postmenopausal women. In women without both diabetes and hypertension,OR of elevated SUA for individuals with vitamin D insufficiency was 2.48 compared to those with vitamin D sufficiency. Moreover, in women with diabetes and/or hypertension, individuals with vitamin D insufficiency were still more likely to have an elevated level of SUA than those with vitamin D sufficiency. However, no significant association between vitamin D insufficiency and elevated SUA was found in each sub-group among premenopausal women.

**Table 2:** Odds ratio of elevated uric acid for women with vitamin D insufficiency by different ages.

Age (years)	25(OH)D	Odds ratio and 95% confidence interval of elevated uric acid			
	(µg/L)	Un-adjusted	*P* value	Adjusted	*P* value
<55	>30	1.00(reference)	-	1.00(reference)	-
	≤30	0.93(0.51–1.71)	0.825	1.00(0.53–1.89)	0.991
≥55	>30	1.00(reference)	-	1.00(reference)	-
	≤30	2.25(1.41–3.58)	0.001	2.38(1.47–3.87)	0.001

Covariates for adjustment include residential site, education status, cigarette smoking, alcohol consumption, total cholesterol, triglycerides, fast plasma glucose, body mass index, systolic and diastolic blood pressure.

### Association between 25(OH)D levels and elevated SUA

Participants were categorized into quartiles of 25(OH)D in premenopausal and postmenopausal women, respectively. Association between 25(OH)D levels and elevated SUA was examined. As shown in Table 3, among premenopausal women, ORs of elevated SUA for individuals in the lower quartiles of 25(OH)D were not significant compared to those in the highest quartile in either univariate or multivariate models. Whereas, a marked association between 25(OH)D levels and risk of elevated SUA was observed among postmenopausal women. In univariate analysis, participants in the lowest quartile of 25(OH)D were more likely to have elevated SUA than those in the highest quartile (*P* = 0.023). As level of 25(OH)D decreased, OR of elevated SUA for individuals in each quartile positively increased (*P* for trend = 0.011). After adjustment for residential site, education status, cigarette smoking, alcohol consumption, TG, FPG, BMI, SBP and DBP, association between 25(OH)D level and elevated SUA remained significant (*P* for trend = 0.008). OR of elevated SUA for women in the lowest 25th percentile of the distribution of 25(OH)D concentration had 2.05 times the OR of elevated SUA than did individuals in the highest 25th percentile (*P* = 0.023).

**Table 3:** Odds ratio of elevated uric acid for women with different 25-hydroxy vitamin D levels.

Age (years)	25(OH)D	Odds ratio and 95% confidence interval of elevated uric acid			
	(µg/L)	Un-adjusted	*P* value	Adjusted	*P* value
<55	≥51	1.00(reference)	-	1.00(reference)	-
	41–50	1.01(0.56–1.82)	0.985	1.04(0.56–1.95)	0.898
	34–40	0.97(0.52–1.78)	0.912	0.08(0.57–2.05)	0.821
	≤33	0.64(0.33–1.26)	0.198	0.66(0.33–1.35)	0.255
	*P* for trend	0.225		0.328	
≥55	≥58	1.00(reference)	-	1.00(reference)	-
	41–57	0.69(0.34–1.41)	0.312	0.65(0.31–1.39)	0.267
	32–40	0.96(0.50–1.83)	0.895	0.98(0.50–1.93)	0.955
	≤31	1.99(1.10–3.60)	0.023	2.05(1.11–3.81)	0.023
	*P* for trend	0.011		0.008	

Covariates for adjustment include residential sites, education status, cigarette smoking, alcohol consumption, triglycerides, fasting plasma glucose, body mass index, systolic and diastolic blood pressure.

## Discussion

In this study, a significant association between vitamin D insufficiency and elevated SUA was found in postmenopausal Chinese Han women. Previous studies suggested a metabolic influence of estrogen on vitamin D and SUA [11], [12], so we studied serum 25(OH)D and SUA levels in premenopausal and postmenopausal women, respectively. We found that the prevalence of elevated SUA was significantly higher in participants with vitamin D insufficiency than those with vitamin D sufficiency (16.50% *vs* 8.08%) among postmenopausal women. Furthermore, the participants were further grouped (shown as Figure 3) according to the status of hypertension and diabetes to eliminate their potential influence on the relationship between vitamin D insufficiency and elevated SUA [16]–[19]. The results showed that the association of vitamin D insufficiency with elevated SUA remained significant in postmenopausal women without both diabetes and hypertension. Some studies have reported that plasma 25(OH)D was associated with the metabolic syndrome [20], therefore, we adjusted for metabolic risk factors such as TG and BMI and other covariates including residential site, education status, cigarette smoking, and alcohol consumption in multivariate logistic regression models. An independent and significant association between vitamin D insufficiency and elevated SUA were found in postmenopausal women. Some studies have demonstrated that supplementation with vitamin D or its metabolites was able to reduce blood pressure in hypertensive patients and blood glucose in diabetics and to improve symptoms of rheumatoid arthritis and multiple sclerosis [11]. However, there is no report about the relationship between vitamin D insufficiency and hyperuricemia which is an important metabolism disorder associated with CVD.

To our knowledge, this is the first study to investigate the association of vitamin D insufficiency with elevated SUA in middle-aged and elderly Chinese Han women. The major strength of this study was that we used the data from a representative sample of women aged above 30 years residing in Suzhou, China. We completed the field study within a relatively short period, which minimized the seasonal variation in the biomarkers. In our analysis, we took into account many potential covariates that might confound the observed association. Additionally, we examined the association between 25(OH)D and elevated SUA in premenopausal and postmenopausal women, respectively. Nevertheless, several limitations should be acknowledged. A cause-effect relationship between 25(OH)D and elevated SUA cannot be inferred because of the cross-sectional nature of the study design. Although data on sun exposure and vitamin D supplementation were not available, we used a direct measure of vitamin D status, which reflected cumulative sun exposure and dietary vitamin D intake. In addition, because we did not measure serum calcium and parathyroid hormone, we could not determine whether the association of 25(OH)D with SUA was partly mediated by calcium or secondary hyperparathyroidism, although individuals with self-reported thyroid or parathyroid disease were excluded. In this study, average menopausal age for Chinese Han women was used to determine whether a participant menopause which may lead to misclassification. As a result, influence of estrogen might not be absolutely eliminated. Further study is needed to assess the association between vitamin D insufficiency and elevated SUA in physiologically postmenopausal women. In addition, data on diet information were not available in our study, so influence of diet on SUA level was not controlled although subjects with SUA lowering treatment were excluded. Renal function, a confounder related to SUA metabolism [6], [21], was not evaluated in our study, although participants with chronic kidney diseases were excluded. We analyzed the association between vitamin D insufficiency and elevated SUA only among females, which maybe affect the extrapolation of the significant association to male population.

Vitamin D insufficiency has been found in chronic kidney diseases [5], [22]. In the kidney, uric acid and urate were initially filtered and additionally secreted. However, the largest part (90%) of secreted uric acid and urate was usually reabsorbed and returned to blood [21]. Thus, impaired renal function can raise circulating SUA concentration by decrease of renal excretion [6]. Meanwhile, vitamin D insufficiency can activate parathyroid to induce the release of parathyroid hormone [9] which was considered to raise SUA level [7], [8], [10], [23], although the mechanism was not clearly explained. Previous clinical trials of postmenopausal women found that parathyroid hormone increased the incidence of hyperuricemia in a dose-response fashion [10], [23]. After cessation of treatment, SUA level returned to or approached pretreatment level [23]. All the findings above support the notion that vitamin D status is inversely associated with elevated SUA. To our knowledge, allopurinol, which blocks uric acid production, is currently the most commonly used drug for lowering SUA level. However, allopurinol may cause adverse events including the rare but potentially life threatening allopurinol hypersensitivity syndrome and its clinical efficacy has not been examined in placebo controlled RCTs [24]. From our findings, vitamin D supplementation may be an alternative therapy for hyperuricemia which has little adverse effects. Therefore, an intensive trial study is needed to determine whether vitamin D supplementation can reduce SUA in hyperuricemia. Recently, in contrast, a small clinical trial [25] which enrolled 192 women ≥65 years and found that the group who received both calcium and vitamin D had significantly elevated concentration of SUA (316.5 umol/L *vs* 291.0 umol/L) compared with those who received placebo.

An increasing body of literature suggested that elevated SUA was a risk factor of CVD [2], [3]. A prospective cohort study [26] of 83,683 Austrian men found that SUA was independently related to cardiovascular mortality, suggesting the clinical importance of monitoring and intervention based on the presence of an increased SUA concentration. However, whether SUA could be a target for treatment in CVD prevention is unknown. Interestingly, a large prospective cohort study [27] of 74,272 women and 44,592 men found that a higher intake of vitamin D was associated with a lower risk of CVD in US men (hazard ratio, 95%CI: 0.84, 0.72–0.97) but not in women (hazard ratio, 95%CI: 1.02, 0.89–1.17). Results from a meta-analysis of 17 prospective studies [28] suggested that vitamin D supplementation at moderate to high doses may reduce CVD risk (pooled relative risk, 95% CI: 0.90, 0.77–1.05). Therefore, the impact of vitamin D supplementation on hyperuricemia and consequently the influence of SUA lowering treatment on CVD prevention are needed to be clarified.

In summary, our findings suggested that reduced 25(OH)D was associated with an increased risk of having elevated SUA in postmenopausal women but not in premenopausal women. Because there is evidence of ethnic and gender variations in the 25(OH)D effect and no data on the association of 25(OH)D with hyperuricemia in Chinese Han women, our data provide novel insights into the nature of this association among Chinese Han women. Moreover, due to the increasing prevalence of hyperuricemia and vitamin D insufficiency in the middle-aged and elderly Chinese population, our results may have important public health implications. Increasing sun exposure and vitamin D supplements are feasible and inexpensive means to prevent vitamin D insufficiency and related health problems. However, the benefits of vitamin D on hyperuricemia need to be confirmed in future prospective studies and clinical trials.
